# Armed Gang Violence in Haiti and the Medication Shortage: Acting Quickly to Save Lives

**DOI:** 10.3389/ijph.2025.1608510

**Published:** 2025-06-23

**Authors:** Jude Mary Cénat, Lewis Ampidu Clorméus, Lukinson Jean

**Affiliations:** ^1^ School of Psychology, University of Ottawa, Ottawa, ON, Canada; ^2^ Interdisciplinary Centre for Black Health, Ottawa, ON, Canada; ^3^ University of Ottawa Research Chair on Black Health, Ottawa, ON, Canada; ^4^ Faculty of Ethnology, University of Haiti State, Port-au-Prince, Haiti; ^5^ Department of African American Studies, Yale University, New Haven, CT, United States; ^6^ Department of Sociology, Campus Henri Christophe-Limonade, University of Haiti State, Port-au-Prince, Haiti

**Keywords:** public health, violence, medication shortage, armed gang, Haiti

Haiti is in a state of collapse. Since 2018, a surge in armed gang violence has caused tens of thousands of direct and indirect deaths, fueling an increasingly alarming health crisis. In 2024 alone, armed gangs killed more than 5,600 people (about 1,000 more than in 2023), with over 1,500 reported kidnappings and thousands of gunshot injuries [1]. Today, more than 85% of the metropolitan area of Port-au-Prince is under gang control, completely isolating the capital and plunging the population into a humanitarian and health catastrophe [2, 3]. Nearly half of the country—5.4 million Haitians—suffer from acute food insecurity, including 1.64 million in critical conditions. Rising prices and widespread shortages are exacerbated by gangs blocking roads and imposing illegal tolls, leaving 65% of households in Port-au-Prince without enough food [4]. Over a million people, half of them children, have been displaced, left without shelter or access to basic services. Violence, famine, and mass displacement have turned the country into a survival zone. Since November 2024, international commercial flights to Port-au-Prince have been suspended, further deepening the crisis by causing severe shortages, including a critical lack of medication (as 70% of medication used in Haiti are imported). In March 2024, the Association of Pharmacists of Haiti (APH) issued a warning that the country’s escalating medication shortage posed a critical threat, with the potential to result in thousands of preventable deaths [5].

Indeed, Haiti’s healthcare system has already collapsed, with fewer than 10% of hospitals operating at full capacity [6]. The January 12, 2010, earthquake had already reduced the capacity of hospitals and the healthcare system to provide necessary care to patients [7, 8]. Most of the country’s major medical centers are now inaccessible, looted, or burned down by armed gangs.

Across the country, persistent gang violence has led to a severe shortage of essential medications, worsening an already fragile healthcare system. This medication shortage endangers emergency care, the treatment of chronic illnesses (e.g., hypertension, diabetes, kidney diseases, cardiovascular diseases, cancer), and the management of infectious diseases (e.g., malaria, pneumonia, tuberculosis, cholera). Hospitals are also suffering from a lack of surgical supplies, antibiotics, and vital treatments for maternal and neonatal care. The crisis has caused a dramatic decline in vaccination rates, increasing the risk of outbreaks of polio, rabies, pneumococcus, and rotavirus. With cholera still circulating, the lack of epidemiological surveillance heightens the threat of an imminent health disaster. Additionally, no psychiatric hospitals are currently operational in the country, leaving individuals with mental illnesses without care, amid a growing shortage of psychotropic medications. The full extent of deaths and complications due to the shortage of medications and medical supplies remains to be assessed. A similar shortage of medication also caused many collateral deaths during the recent civil war in Syria [9]. Indeed, as demonstrated during the conflict in Syria, prolonged conflicts weaken healthcare systems and exacerbate medication shortages, leading to preventable deaths.

In February 2025, the Pan American Health Organization (PAHO) delivered 7.7 tons of medications to Haiti to support some hospitals, provide limited vaccines, and offer temporary relief from the shortage [6]. However, without a rapid and large-scale response to the medication crisis, the country risks falling into an even more dire health situation, with more deaths, more unvaccinated children, and more irreversible health conditions.

To mitigate the medication shortage, reduce related deaths, and alleviate the suffering of local populations, an urgent multisectoral public health approach is necessary (see Table 1). The Ministry of Public Health and Population (MSPP), in collaboration with regional health directorates, must urgently develop a comprehensive mapping of medication needs, considering various disease groups, emergency medical interventions, preventive measures, and regional disparities. The World Health Organization (WHO) and the PAHO should provide technical support to accelerate the development and dissemination of this plan. Additionally, Haitian researchers and healthcare professionals from the diaspora could contribute their expertise to create a needs assessment map within 10 days through an efficient task force.

**TABLE 1:** Public health emergency strategic plan to mitigate the medication shortage in Haiti (Port-au-Prince, Haiti).

Strategy	Implementation actions	Involved actors
Immediate interventions		
Needs assessment and mapping	Map out the medication needs, considering cross-cutting and specific needs in the 10 geographic departments of Haiti	Task force involving, MSPP, regional health directorates, Haitian Medical Association (AMH), Haitian Pharmacists Association (HPA). Supported by PAHO/WHO. Supported by Haitian researchers and healthcare professionals living abroad
Urgent global call for medication donations	Launch a global emergency appeal for medication donations to support Haiti for 6 months to 1 year (from countries, international agencies, NGOs, including those from the Haitian diaspora)	Launched by WHO, PAHO, and the UN Secretary-General’s office
Medication quality control	Reject medications close to expiration and experimental treatments; suspend clinical trials	PAHO should establish an expiration delay for each medication group, in discussion with the HPA
Elimination of Customs Barriers	Remove administrative and customs barriers to allow MSPP to receive supplies under the supervision of WHO and PAHO	MSPP/ Ministry of Commerce
Secure supply chains	Set up a secure airbridge to transport medications to Haiti	National and international security forces present in Haiti
Free Distribution	Ensure free access to medications and vaccines, particularly for children aged 0 to 5	MSPP and regional health directorates
Medium-Term Actions		
Medical Deployment	Set up temporary clinics and mobile teams for chronic care and emergencies	MSPP and regional health directorates
Establish humanitarian corridors	Collaborate with security forces to secure supply routes beyond the crisis	MSPP, regional health directorates and National and International Security Forces Present in Haiti
Drone Delivery of medication	Use drone technology for hard-to-reach areas	MSPP/Ministry of the Interior
Long-Term Actions		
Local Pharmaceutical Production	Develop national capabilities for pharmaceutical manufacturing to reduce dependence on imports	MSPP, Private Sector
Healthcare sector funding	Increase the MSPP budget beyond its current level (<2% of the national budget of under 2 billion USD) to strengthen healthcare infrastructure	Government of Haiti

Simultaneously, WHO, PAHO, and the United Nations Secretariat should launch a global appeal for an emergency six-month supply of essential medications. Strict guidelines must be enforced to reject nearly expired medications and experimental treatments, while all ongoing clinical trials should be suspended to ensure patient safety. Eliminating customs formalities for receiving medical supplies is crucial for a period of 6 months to a year, with deliveries received directly by the MSPP under the supervision of WHO and PAHO.

A secured airbridge, supported by law enforcement and commercial transport companies, should facilitate the rapid and safe delivery of supplies, ensuring the cold chain is maintained and that medical care continues in different regions according to their specific needs. Given the widespread impoverishment of the Haitian population, medications and vaccines, particularly for children aged 0 to 5, must be provided free of charge, alongside measures to involve pharmacies in distribution and ensure they receive financial compensation.

Beyond emergency measures, sustainable actions must be implemented to strengthen the resilience of Haiti’s public healthcare system. The public health response should also include the launch of a national vaccination campaign to prevent outbreaks of vaccine-preventable diseases, prioritizing children aged 0 to 5. In the medium term, to ensure access to healthcare—including in rural areas—the deployment of temporary clinics and mobile medical teams is essential for treating chronic illnesses and non-hospital emergency cases. Integrating drone technology for medication distribution in remote areas could also improve logistical efficiency.

In the long term, Haiti must prioritize local pharmaceutical production and significantly increase the healthcare sector’s budget, which currently represents less than 2% of the national budget (with a total budget of under two billion USD for a population of over 12 million), prioritizing public health policies. These measures should help reduce dependence on foreign aid and build a resilient public healthcare system. Investments must also be made in decentralized storage infrastructure that meets international standards for proper medication conservation, while ensuring the implementation of a computerized tracking and management system to prevent shortages and waste.
